# PRODIS: a proteomics data management system with support to experiment tracking

**DOI:** 10.1186/1471-2164-12-S4-S15

**Published:** 2011-12-22

**Authors:** Alessandra Faria-Campos, Herbert Fernandes-Rausch, Celina Val, Peter Thorun, Vinicius Abreu, Paulo Henrique Batista, Paulo Henrique Mendonça, Vinicius Alves, Maíra Ribeiro Rodrigues, Adriano Pimenta, Glória Franco, Sérgio Vale Aguiar Campos

**Affiliations:** 1 Departamento de Ciência da Computação, Universidade Federal de Minas Gerais, Brazil; 2 Departamento de Bioquímica e Imunologia, Instituto de Ciências Biológicas, Universidade Federal Minas Gerais, Brazil; 3 Departamento de Biologia Geral, Instituto de Ciências Biológicas, Universidade Federal Minas Gerais, Brazil

## Abstract

**Background:**

A research area that has greatly benefited from the development of new and improved analysis technologies is Proteomics and large amounts of data have been generated by proteomic analysis as a consequence. Previously, the storage, management and analysis of these data have been done manually. This is, however, incompatible with the volume of data generated by modern proteomic analysis. Several attempts have been made to automate the tasks of data analysis and management. In this work we propose PRODIS (Proteomics Database Integrated System), a system for proteomic experimental data management. The proposed system enables an efficient management of the proteomic experimentation workflow, simplifies controlling experiments and associated data and establishes links between similar experiments through the *experiment tracking* function.

**Results:**

PRODIS is fully web based which simplifies data upload and gives the system the flexibility necessary for use in complex projects. Data from Liquid Chromatography, 2D-PAGE and Mass Spectrometry experiments can be stored in the system. Moreover, it is simple to use, researchers can insert experimental data directly as experiments are performed, without the need to configure the system or change their experiment routine. PRODIS has a number of important features, including a password protected system in which each screen for data upload and retrieval is validated; users have different levels of clearance, which allow the execution of tasks according to the user clearance level. The system allows the upload, parsing of files, storage and display of experiment results and images in the main formats used in proteomics laboratories: for chromatographies the chromatograms and lists of peaks resulting from separation are stored; For 2D-PAGE images of gels and the files resulting from the analysis are stored, containing information on positions of spots as well as its values of intensity, volume, etc; For Mass Spectrometry, PRODIS presents a function for completion of the mapping plate that allows the user to correlate the positions in plates to the samples separated by 2D-PAGE. Furthermore PRODIS allows the tracking of experiments from the first stage until the final step of identification, enabling an efficient management of the complete experimental process.

**Conclusions:**

The construction of data management systems for Proteomics data importing and storing is a relevant subject. PRODIS is a system complementary to other proteomics tools that combines a powerful storage engine (the relational database) and a friendly access interface, aiming to assist Proteomics research directly at data handling and storage.

## Background

A research area that has greatly benefited from the development of new and improved analysis technologies is Proteomics - the process of identification and quantitative analysis of proteins expressed in different conditions or life stages of a cell or organism [1]. To analyze a complex sample in terms of its protein contents it is often necessary to perform a series of experiments, generating a comprehensive and complex amount of data. Proteomic analysis encompasses the use of several technologies such as liquid chromatography (LC), bi-dimensional electrophoresis (2D-PAGE) and mass spectrometry (MS). Each uses specific instruments performing the main stages of the analysis and generating large amounts of data. Modern spectrometers for example, can generate over a gigabyte of compressed binary data per hour [2]. The data produced by the different instruments usually differ in type and structure, thus making integration of so diverse information a special challenge. Given the nature and relevance of this data, the main challenge nowadays is how it can be stored with minimum human intervention, integrated and treated in a way that allows its dissemination and mining in a efficient and productive way [3]. Some solutions have been proposed to help addressing this problem, including the use of Laboratory Information Management Systems (LIMS) and databases designed especially for Proteomics. Most existing Proteomics databases are usually related to only one type of data and/or represent already processed results, not raw data. Therefore, Proteomics researchers frequently have to resort to several different data repositories in order to be able to gather all information needed to perform the complete identification and characterization of a protein [4,5].

Because there was little integration between experimentation, analysis and comparison with existing data, the experimental unprocessed data produced was usually stored without any means to associate it automatically with results of analyses and resulting publications. However, several initiatives including those associated with the Human Proteomics Organization (HUPO) have discussed the importance of unprocessed raw data availability in association with processed data and the need for unified databases harboring both types of data [6-9]. Thus, specialized systems have been proposed aiming to fulfill this need. To accomplish this task, these systems have to fill some basic needs of proteomic research: (i) storage of raw data, images from 2D-PAGE and peak lists obtained from LC and MS, (ii) storage of experimental parameters, data sets and results and (iii) analysis of quantitative and non-quantitative data. The LIMS available for Proteomics nowadays aim to meet these needs and open source and proprietary systems can be found such as LIPAGE, CPAS, BioinformatiQ, Mascot Integra and ms_lims [3,9,10].

Some initiatives have been aimed especially towards unprocessed and raw data management. Among these are MASPECTRAS and Proteios - systems for data management and the PRIDE database - a repository for protein and peptide identifications [9,11]. MASPECTRAS is a web-based system designed for the management of mass spectrometry data with some functionalities towards the analysis of this data, integrating search engines and tools that allow comparison of multiple searches [12]. The Proteios system, was initially developed as an open source system for storage, organization, analysis and annotation of Proteomics experiments and is now part of the ProSE, an analysis platform targeted for multiple users that integrates management and analysis of the data with a programming interface that enables local extensions and database access [13,14]. The PRIDE system is a structured data repository that stores three different kinds of information: peptide and protein identifications derived from MS or MS/MS experiments, MS and MS/MS mass spectra as peak lists and all associated metadata [15]. PRIDE is an important repository to which data can be submitted through a web interface, using either the PRIDE XML schema, which embeds mzData as a sub-element to allow inclusion of details of the spectra, or using the mzData XML schema. Searches can be made also using the same data formats, allowing the user to retrieve data on protein identifications on XML or human-readable formats [11,16]

These solutions while very important and complementary, lack still some functionalities to allow a complete exchange and comparison of data from different experiments used in Proteomics laboratories. In order to help fulfill the needs for these functionalities, we propose in this work the Proteomics Data Integrated System (PRODIS), a system designed for management of experimental data from Proteomics experiments. The system manages experiment information, unprocessed data and analysis results from LC, 2D-PAGE and MS experiments. It’s main objective is to offer a simplified interface to allow the tracking of all relevant information to an experiment including images and associated files generated by analysis instruments. It provides basic project management functionalities and works as an experimental data repository extended to automate the submission of data to protein identification tools such as Mascot, X-Tandem and OMSSA. Moreover, PRODIS not only stores the data, but also provides *experiment tracking*, which relates associated experiments allowing them to be retrieved together or individually. This makes it possible, for example, to track samples from the initial protein extraction experiment until the MS identification. Furthermore, it is possible to track results from intermediate experiments, not only from the initial sample. For example, it is possible to identify which spot in a gel originated a specific *m/z* list. This makes it simpler to identify patterns such as similar experiment parameters that could only be clearly identified after the individual analysis of a series of experiments. Experiment tracking increases the efficiency of the experimental process by uncovering hidden relationships between experiments and/or samples that can assist in protein identification, but could have been overlooked otherwise.

## Implementation

PRODIS has been implemented on the server using Apache web server and a MySQL server version 5.0 running over a Pentium 2.5 GHZ machine using Linux Suse distribution 11.0. The database construction uses a relational approach and data indexes to associate projects to experiments, the experiments to their information and results and to each other. The PRODIS database is accessed using a browser through a web based interface that uses PHP scripts to communicate with the database. Experimental data resulting from analysis aided by instruments are inserted in the database using the web interface as well with the help of parsers that interpret the data directly from files generated by the instruments used in the analysis. Perl scripts are used for submission of *m/z* files to identification tools installed on the server (X-Tandem and OMSSA) or accessed remotely (Mascot).

### PRODIS data model and scope

**Figure 1:**
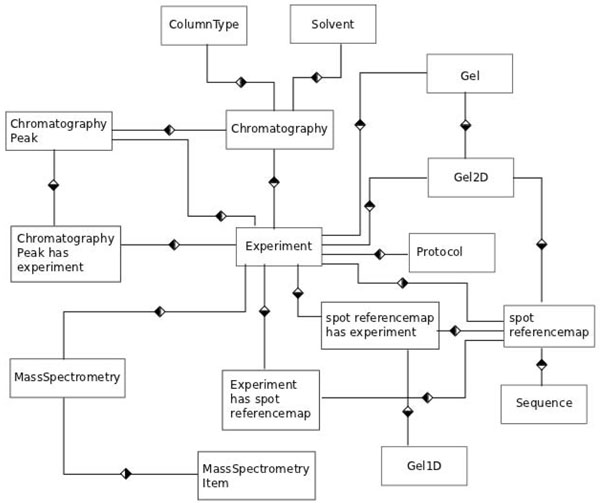
PRODIS data model.

Proteomic analysis can be performed using a diversity of experimental setups. One of these is the use of a separation platform such as 2D-PAGE and/or LC followed by a run in an identification platform that uses MS. A data model designed for a Proteomics data management system has to take this into consideration in order to represent the sequence of experiments correctly. PRODIS data model has been designed considering these needs and includes three main groups of tables: (i) those designed to store general information on projects such as name, description, coordinator, members, associated publications, etc; (ii) those designed to store protocols and experimental conditions; and (iii) those designed to store results and result-associated files. A simplification of the data model can be seen in Figure 1, the full model has 46 tables and can be downloaded from the PRODIS web site.

PRODIS maintains two types of information, the conditions under which an experiment has been performed and its results. Experimental conditions stored include identification of samples, solvents, temperature, instrument used for the analysis, etc. Experiment results stored are different for each type of experiment. For 2D-PAGE, PRODIS stores gel images and Image Master Platinum (GE HealthCare) files; for LC peak lists are stored, and for MS *m/z* lists and protein identification files are stored in the database. The PRODIS data model has the experiment as central entity and handles three types of data: LC, 2D-PAGE and MS. For each type of experiment there is a set of tables associated to it and entries associated with the experiment. These entries store the experimental conditions, the protocol used to perform it, one or more specific results (a list of chromatography peaks, images of gels, *m/z* lists, etc.) and all the information regarding the project to which it belongs. Images and chromatogram files are stored outside of the database, with links to these files stored in the database along with all results. As a result of its design, in PRODIS, the graphics (chromatograms or gel images) are directly associated to the experiments and the specific samples used in a prior stage of the experiment, making easy to track and control all steps executed up to protein identification.

**Figure 2:**
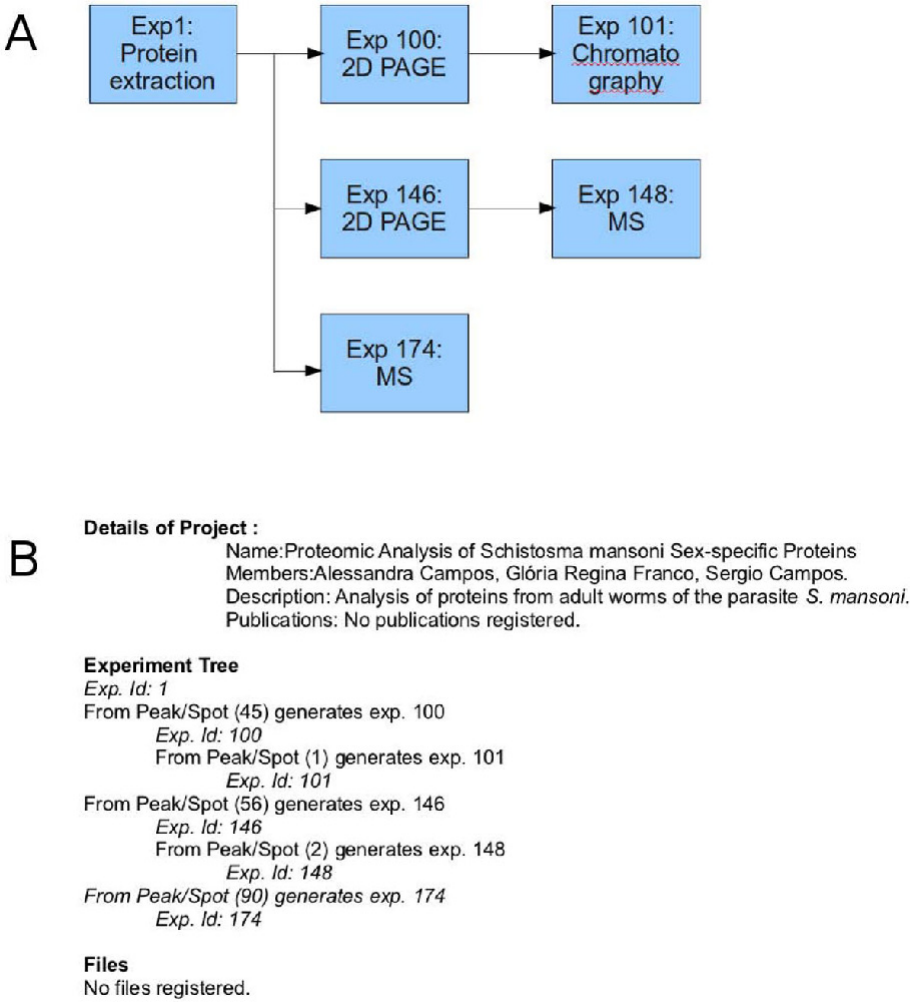
Experiment tracking in PRODIS through the experiment tree present in reports. A. Example of a set of associated experiments. B. Experiment tree generated in the report of Experiment 1.

The experiment is the main component of the model because PRODIS is aimed primarily at assisting researchers in tracking experiments and cross-linking experimental data. The data flow starts when a new experiment is performed and its information is entered in the *experiment* table. Each experiment receives an internal ID that identifies it uniquely. According to the type of experiment the tables associated to that type of experiment are also filled and associated to the internal ID. Experiments are often related to other experiments, such as when a particular result prompts the researcher to perform a more detailed analysis on a peak or spot. PRODIS stores the associated experiments by cross-linking the internal IDs which allows it to establish a temporal link, so that the sequence of experiments performed can be followed later. This complex set of operations can be visualized by the user as an experiment tree in reports produced by the system (Figure 2B).

The data model proposed has been designed using as model data from the proteomic analysis of the centipede *Scolopendra viridicornis* (LC) and the parasite *Schistosoma mansoni* (2D-PAGE and MS). Final analysis of these data is available on [17] and [18].

### Experiment tracking

The main feature of PRODIS developed to help improve proteomic analysis is the ability to track all steps used in the experimental process, from sample extraction to protein identification. This feature, known as *Experiment tracking*, is implemented in PRODIS by associating related experiments in the database through the use of ids to tag these related experiments. When an experiment is inserted in the system the user has the option of associating it with an existing experiment. This generates what is called in PRODIS an *experiment tree*, which contains the relationship between experiments. In this way it is possible to identify exactly how experiments are related to one another, assisting the researchers in controlling the flow of experiments performed, the samples used and also in explaining the results and how they were obtained In order to identify which sample has originated a given result, one can simply look for the first experiment in the experiment tree of the experiment being analyzed. The first experiment data will contain the information on the sample that has been used in the analysis. In a similar way, given a specific sample which has been used in an experiment, the experiment tree for it will identify all experiments that use this sample. Experiment tracking can provide even richer information, however. It is possible to identify, for example, which spot in a given gel has originated a certain *m/z* list. The experiment tree for the specific *m/z* list provides this information.

The experiment tree is not tied to a certain type of experiment. It can identify related experiments from any given experiment. In this way the researcher can trace back or forward from any point establishing the causes or consequences of any step in the experimental process, helping to correct problems in the experimental process, or improve it by uncovering relationships that are hidden in the data.

**Figure 3:**
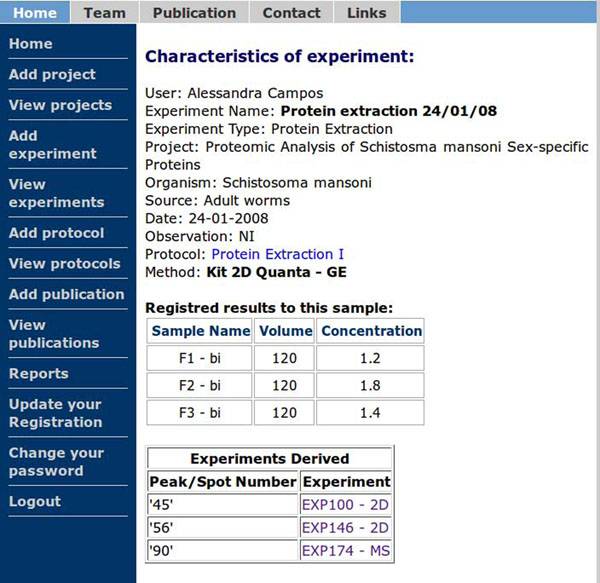
Experiment tracking in PRODIS through the View experiments option.

Figures 2 and 3 illustrate how the experiment tree can be used in PRODIS. At this example, samples obtained in Experiment 1 (protein extraction) were used in Experiments 100, 146 and 174. The resulting products are used in Experiments 101 and 148 (Figure 2-A). After finishing the experiment, a report containing the experiment tree is generated (Figure 2-B), where the above relationships are described. The experiment tree shows which experiments are related to each other and in which order. As any experiment details or results can be consulted through the *View experiments* option in PRODIS, the experiment tracking can be also performed by using this option. When using the *View experiments* option all data related to the experiment can be seen. In the bottom of the page, PRODIS shows the experiments that are parents and children of the current experiment. Figure 3 shows the information using the *View experiment* option for Experiment 1. On the bottom of the page can be seen the experiments generated from Experiment 1 : Experiments 100, 146 and 174. By clicking in the link with the experiment name the user can navigate the experiment tree and efficiently locate the experimental data needed.

### Submission and data retrieval

**Figure 4:**
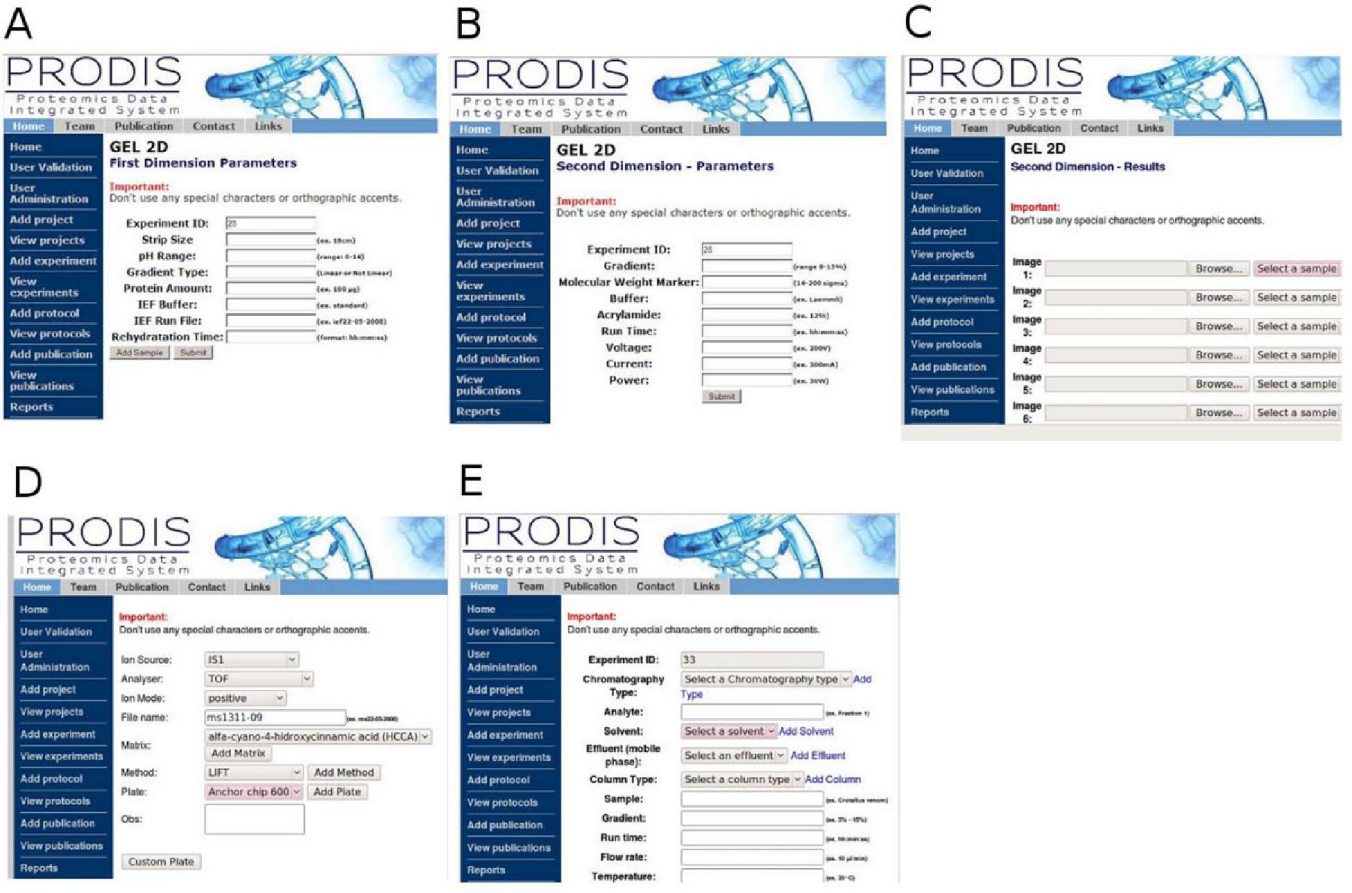
PRODIS interface for data submission. A. 2D-PAGE parameters and conditions - first dimension; B. 2D-PAGE parameters and conditions- second dimension; C. 2D-PAGE image and file upload screen; D. Mass spectrometry -parameters and conditions; E. Liquid Chromatography parameters and conditions.

PRODIS is designed to store general information on projects and experiments, LC data from the AKTA Explorer 100 (Amersham BioSciences) HLPC system, 2D-PAGE data generated using the Image Master 2D-Platinum system (GE HealthCare), *m/z* files from MS (mzXML, pkl, mzML and mzData formats) and identification files from Mascot, X-Tandem and OMMSA runs. At the moment PRODIS has been designed to small Proteomics facilities and does not present functionalities for on-line LC-MS setups. The screens to collect data for this kind of setup are currently being developed. Data can be submitted to PRODIS through the web interface in a friendly and intuitive way. PHP scripts were designed to upload the data inserted through forms in the database and Perl scripts parse all the files uploaded inserting data in the database or directing files to the proper directory (Figure 4). A series of drop-down menus have been included in the interface as a way of minimize user typos and decrease errors in data upload. Data retrieval in PRODIS is also performed through the web interface. Data can be viewed as human-readable HTML or reports in printable format can be retrieved from the system.

### MIAPE compliance

Several initiatives including those associated to the HUPO have discussed the importance of a set of standards for proteomics data publication. Therefore, MIAPE has been developed by the Proteomics Standards Initiative of HUPO (HUPO-PSI) as a set of guidelines representing the minimal information required to report and sufficiently support assessment and interpretation of a proteomics experiment [19]. PRODIS data upload functions includes all information demanded by MIAPE. The fields for uploading information required by MIAPE are spread throughout the several scripts of the interface. For submission to other databases or publication, these data can be exported by the user directly from the database in PDF format. An XML format exporter is being finalized.

### Data access in PRODIS

Data access is controlled through a password protected system in which each screen for data upload and retrieval is validated. Users have different levels of permission, which allow the execution of tasks according to the user profile. This approach allows different projects to be registered in the system and use it without any risk of data sharing or leakage between them. PRODIS accomplishes this task through a comprehensive protection mechanism that associates users with the tasks they performed, and also tasks and users with managers that can oversee experiment order with ease and reliability. Each user in PRODIS is identified in the system through its login/password, and is given a set of permissions. Permission levels vary from guest, to coordinator. Guests can only access a limited set of experiment data, while coordinators can access and update all data associated with its projects. There are also permission levels associated with project membership. A researcher can access and update data from its own experiments, see data associated with experiments of the same project, even if inserted by other project members, and cannot see data that belongs to projects to which they do not belong. In this way it is possible to track experiments not only by storing all information related to the experiment but also by ensuring that each researcher has the appropriate permissions to execute the experiment, and by maintaining the information about who performed which experiment. By entering experiment information in the system all this information is automatically recorded, so researchers can easily identify which experiments they did, and analyze their results. Also, project managers can see which projects members executed which parts of the analysis and how their work progresses as the project is developed.

## Results and discussion

The amount and diversity of data generated at Proteomics experiments is very large no matter which workflow is used. The use of automated systems to handle and treat such data is an important issue on Bioinformatics today. Several systems have been developed with that goal. However, few of those allow the registering of experimental conditions in all the steps used for protein identification. In this paper we present PRODIS, the Proteomics Data Integrated System, a Proteomics integrated data management system designed to store unprocessed raw and analyzed data for a Proteomics workflow that allows experiment tracking along with project management. The experiment tracking feature connects the information produced by different types of experiments in order to be able to present to the researcher a picture of the experimental process with all steps and conditions used in the process. This feature makes it easier to access all data related to each other and to specific experiments increasing the reliability of the analysis.

The focus of PRODIS is to manage and store unprocessed data and experimental conditions as well as to help researchers in tracking data generated by individual experiments and how this data relates to other experimental data even before the experiment results have been completely processed. As it was designed to manage rather that analyze data, PRODIS is a system complementary to other Proteomics tools such as PRIDE, ProSE and MASPECTRAS. These systems manage the data regarding proteomic identification focusing especially on mass spectrometry and protein identification. PRODIS main concern is the storage of experimental data and the information on how the experiments have been performed regardless of the experiment type.

The scope of PRIDE is mainly the information regarding peptide and proteins identified by the user and the data and metadata associated with it such as the sequence and coordinates of the peptide within the protein that it provides evidence for, any post-translational modifications coordinated in relation to the specific peptide that they have been found upon, instrumentation used to perform the analysis and processed peak lists supporting the identifications. PRODIS on the other hand works as a system complementary to PRIDE storing the general information concerning the conditions and protocols used in the identification experiments since sample extraction, providing a snapshot of the experiments performed and their relationships, in order to show the most successful experimental conditions and point possible mistakes not only for MS experiments but also for the other steps in the proteomic analysis.

ProSe is a system similar to PRODIS as it handles LC, MS and 2D-PAGE data. ProSe supports the sample tracking function. However, sample tracking only allows one to see which experiments have been generated from a given sample. It is not possible to associate all related experiments as allowed by the experiment tree constructed by PRODIS. Using PRODIS it is possible to identify experiments that are related to *any* experiment performed, not only the initial sample. For example, it is possible to identify which spots generated a *m/z* list on a MS experiment and all the data associated to it such as gel images or files. The main strength of PRODIS is that it is a system to help the researcher at the initial steps of proteomic analysis by storing experimental data and experiments attributes independent of which protein it relates to and allowing all information to be retrieved regardless of the step of analysis being performed.

MASPECTRAS focuses on mass spectrometry, and PRIDE on protein identification. Even though some functionality exists in these systems for managing other types of experiments, PRODIS makes it simpler and more efficient to track the complete Proteomics experimental process. ProSe handles more types of experiments, but its sample tracking functionality is not as comprehensive as PRODIS experiment tree. PRODIS uses a web server as the interface for data capture. Simple forms constructed in the PHP language are made available for data entry. The researcher uploads the result files generated directly from the experiment using this interface. The files are processed through parsers that are used to interpret this data directly out of the experiment results. Using a web based interface gives an increased portability to data capture since users do not need to install any specific software to have access to the database for data importing and exporting. Also, this system makes data importing and storing faster and less error prone than a manual input, since the files are imported automatically, processed by the parsers and inserted directly on the database, without user interventions such as typos or wrong naming of some attributes. This is also an advantage over ProSe, which has a complex interface, and even after installation may require the installation and use of “plugins” to perform some of its functions. Using ProSe efficiently must be preceded by a installation and/or customization step which requires technical expertise. PRODIS, on the other hand, even though less flexible, is simpler to use. After installation, which consists of copying the PHP scripts to a web server accessible directory, accessing a web page is all that is required.

PRODIS has been designed to store data from experiments of several different organisms. At the moment data from *S. viridicornis* and *S. mansoni*, proteomic studies are being collected to feed the database. These experiments have been performed in different laboratories by different research groups, demonstrating the usefulness of the PRODIS, which can provide assistance in the Proteomics research for a large scientific community. Moreover, it demonstrates the capability of the database to store data from different formats and research groups, emphasizing also its flexibility.

## Conclusion

The construction of data management systems for Proteomics data importing and storing is a relevant subject. We have developed a tool that combines a powerful storage engine (the relational database) and a friendly access interface, aiming to assist Proteomics research directly at data handling and storage. The next features to be added to the PRODIS system will be a set of tables containing transcriptomics information such as microarray results and ESTs sequences and direct links to public Proteomics databases. The implementation of theses feature will make the system a more complete tool providing access to several kinds of information and leading to a more complete proteomic analysis.

## Availability

PRODIS is a fully web based system and can be accessed at the URL: http://syrah.luar.dcc.ufmg.br/prodis. A Guest account is set up with login guest and password guest. On this page there is also a link to allow registration of new users.

PRODIS is based on Linux, PHP and MySQL, and is currently in beta testing. A link to download its source code is available at http://syrah.luar.dcc.ufmg.br/prodis.

## Competing interests

The authors declare that they have no competing interests. 

## Authors' contributions

• Alessandra Faria-Campos is the main author and is responsible for the initial design of the system, its data model, supervising its development and for training the developers and users of the system. She is has also performed several of the Proteomics experiments that were used as tests in the system.

• Herbert Rausch is the main developer of the system.

• Celina Val, Peter Thorun, Vinícius Abreu, Paulo Batista and are computer science students that contributed for the development of the system.

• Maira Ribeiro, Vinicius Alves and Paulo Mendonça are responsible for the construction of parsers and installation of protein identification tools.

• Adriano Pimenta and Glória Franco contributed with data from *S. viridicornis* and *S. mansoni* analysis, as well as discussions on how to store and manage Proteomics data in PRODIS.

• Sérgio Campos has assisted with the design of the system and with its internal architecture. He has also supervised the developers and participated in the development of the data model.
